# Guided Periodontal Surgery: Association of Digital Workflow and Piezosurgery for the Correction of a Gummy Smile

**DOI:** 10.1155/2020/7923842

**Published:** 2020-04-08

**Authors:** Tatiana Miranda Deliberador, Suyany Gabriely Weiss, Alexandre Teixeira Domingues Neto, Isabela Zago Zetola, Maria Eduarda Santana Prix, Darlan Rigo Júnior, Heloysa Hoffman Martins, Carmen Lucia Mueller Storrer

**Affiliations:** School of Health Sciences, Positivo University, 5300 Professor Pedro Viriato Parigot de Souza Street, Campo Comprido, Curitiba, PR, Brazil 81280-330

## Abstract

Digital flow has become a part of currently practiced dentistry. Virtual planning ensures predictable aesthetic and functional rehabilitation, painless postoperative recovery, and better communication with patients, thus meeting their expectations. The purpose of this case report is to demonstrate the digital planning for the correction of a gummy smile with a personalized preparation using a piezoelectric surgical guide (PerioGuide) for gingival contouring and flapless osteotomy. The guide was designed using Nemo Studio software, based on the patient's facial aesthetic analysis, through photos, videos, and facial scanning. These images were aligned with the scan and placed over the cone beam computed gingival tomography for prediction of results, based not only on the distance from the cementoenamel junction to the bone crest but also on the best gingival margin contour according to virtual aesthetic planning. Digital planning, combined with the use of a piezoelectric device, allows for a flapless guided surgical technique for gingival contouring and osteotomy. As a result, the surgical procedure is safer, faster, and more predictable with better postoperative outcomes.

## 1. Introduction

A perfect smile is dictated by a balance between three parameters: teeth, gum, and lips. Excessive gingival display while smiling is a concern for many patients, affecting both esthetics and psychosocial behavior [1]. Excessive gingival display, also described as “gummy smile” (GS), has been observed in 7% of men and 14% of women in the age group 20–30 years [2].

GS has several etiologies, such as vertical maxillary excess, anterior dentoalveolar extrusion, altered passive eruption, lip length and activity, and over compensatory eruption, among others, and although there are distinct causes, it is often the result of a multifactorial effect [3–5]. An accurate differential diagnosis is crucial for proper therapeutic application, as the treatment of a gummy smile includes facial surgery, periodontal surgery, laser, and botulinum toxin injection. More recently, digital workflow and flapless surgery (piezosurgery), among others [1, 6, 7], have been associated with treatment planning.

Given the growing demand for aesthetics and less invasive techniques, the digital workflow concept allows better communication with patients, offers the clinician a diagnostic tool to make the most appropriate therapeutic choice, and enables accurate treatment planning with predictable results [8]. Digital treatment planning is based on diagnostic methods such as cone beam computed tomography for the analysis of hard and soft tissues. When the buccal mucosa and the tongue are retracted from the gingival tissues, the images produced allow visualization of the facial and lingual mucosa, determination of the size of the gingival unit (gingival phenotype), and assessment of the ideal relationship between hard and soft tissues for surgical planning of gummy smile correction. The ability to determine the need for osteoplasty increases the accuracy and predictability of the surgical procedure [6, 9]. Moreover, facial scanning may also be used to evaluate digital photographs and intraoral scanning within the digital workflow. With the facial scan information integrated with data, obtained from intra- and extraoral scanners, the treatment design is more likely to reflect the aesthetic demands imposed by the patient [10]. Moreover, piezoelectric bone surgery overcomes the limitations of traditional instrumentation in oral bone surgery, allowing greater intraoperative control and safe cutting [11].

The purpose of this study is to report a clinical case of guided periodontal surgery for the treatment of a gummy smile using digital planning and piezosurgery.

## 2. Case Presentation

**Figure 1:**
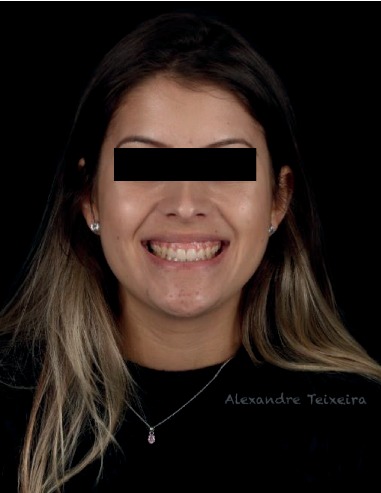
Initial smile.

**Figure 2:**
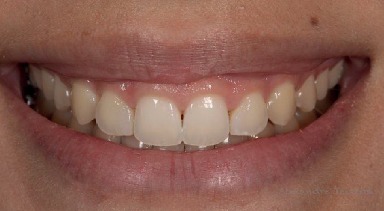
Initial smile, approximate view.

A 19-year-old woman (M.K.S.) with leukoderma sought clinical service dissatisfied with the aesthetics of her smile due to excessive gingival exposure (Figures 1 and 2). Clinical examination revealed short maxillary anterior teeth with excess gingival tissue. Periodontal examination revealed probing depths 2–3 mm with adequate levels of periodontal health (no plaque, bleeding, or periodontal pockets). After clinical and periodontal examination, previous photographs, intraoral scans, and facial scans were obtained. Tooth-gingival computed tomography was requested, and the patient signed a consent form.

### 2.1. Digital Planning

Digital treatment planning begins with an analysis of digital photographs, intraoral scanning (STL) images (3Shape, Copenhague K, Dinamarca), facial scanning (OBJ) (Done 3D, Ribeirão Preto, São Paulo), and tomography images, performed to delimit all dental faces, in addition to soft and hard tissues. Three-dimensional images are loaded in the NEMOTEC program (Nemotec, Madrid, Spain) in which the STL model is aligned with the patient's initial photos. Lines are drawn to mark the symmetry of the face and tooth-gingival proportion in the face-guided intraoral planning. The interpupillary line which establishes the horizontal plane should be parallel to the incisal line and the gingival margin contour. The midline of the face is determined by tracing the glabella, nose, filter, and chin to enable an analysis of facial symmetry. If any deviation is noted, a previous treatment (orthodontics, orthognathic surgery, and prosthetic) may be indicated. The incisal line is a horizontal tracing over the lower lip that facilitates the initial evaluation between the facial lines with the smile. The smile zenith curve is to be demarcated: the canine should be 0.5 to 1 mm higher than the central incisors and the lateral incisors 0.5 to 1 mm lower than the central incisors. The position of the interdental papillae must be beyond the interproximal contact point, closing the interdental space, to promote an aesthetically balanced smile. Finally, the aesthetic aspect ratio, height × width, of the latter elements is verified. The size of the central incisors is based on the ideal proportion to provide a pleasant aesthetic smile, between 9.5 mm and 11 mm (Kurbad et al., 2013).

**Figure 3:**
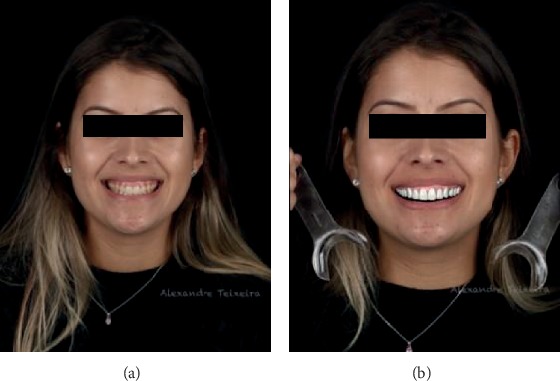
(a) Initial smile. (b) Digital waxing.

Based on the face-guided intraoral planning analysis, digital wax-up was prepared (Figure 3). A correlation was made between the facial shape and the shape of the teeth (square, ovoid, or triangular). Above all, one should always plan a smile that fits the patient's desire, physical appearance, and personality, providing an aesthetic and functional result. After the digital teeth wax-up was completed, the facial scan was juxtaposed with the initial photos of the case (3D Planning) to ensure that the newly acquired positions would be harmonious for the patient's face.

The digital wax-up was printed on acrylic resin (PrintedMockup BDS, Curitiba, Brazil) for patient approval. It was then fixed in the mouth with the aid of flow A1 composite resin (Yller, Pelotas, Brazil). Next, new photographs and videos of the patient's case with the mockup were taken.

### 2.2. Periodontal Profile Analysis

3D digital planning was performed with the Nemo Studio program (Nemotec, Madrid, Spain). Through this feature, it was possible to estimate the ideal amount of gingival tissue to be removed in each dental element to achieve a more aesthetic gingival contour. The measurement obtained through digital planning was superimposed on the images acquired in the dental gingival tomography to observe the relationship between biological distances and define the best surgical approach.

In the Nemo Studio program, the tomography contrast change (threshold) is made for better visualization of hard tissues. STL overlap with DICOM (cone beam tomography) is performed to delimit the amount of soft and hard tissue that will be removed. The accuracy of the surgical guide is increased through this alignment as tomography facilitates visualization of the distance from the cementoenamel junction to the bone crest and from the gingival margin to the cementoenamel junction in millimeters. Moreover, clinical and radiographic examination revealed a diagnosis of altered passive eruption.

### 2.3. Periodontal Surgical Guide

**Figure 4:**
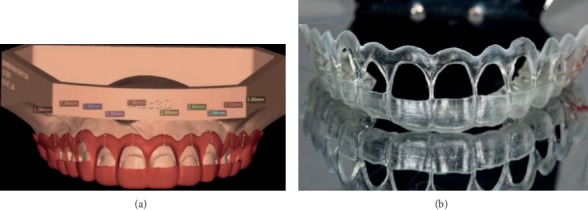
(a) Periodontal analysis and digital planning of the guide. (b) Printed surgical guide.

**Figure 5:**
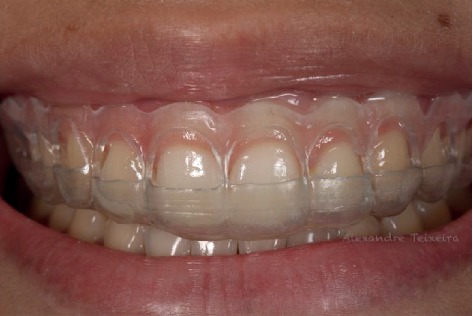
Surgical guide in the mouth. The superior contour of the guide denoted a measurement of 3.0 mm above the new position of the gingival margin defined by the aesthetic waxing.

The surgical guide was designed in the same software, according to the waxing of the new gingival margin position, and printed on resin (PerioGuide) (Figures 4(a) and 4(b)), with 1 mm thickness. The superior contour of the guide denoted a measurement of 3.0 mm above the new position of the gingival margin defined by the aesthetic waxing, aiming to reestablish biological distances (Figure 5). In the “windows” of the guide, a cut was made for the design of the new regular concave arch gingival margin. The purpose of this cutout was to establish the exact limit for gingival tissue incision, avoid excessive tissue removal, and establish the ideal contour determined by the treatment plan.

### 2.4. Surgery

**Figure 6:**
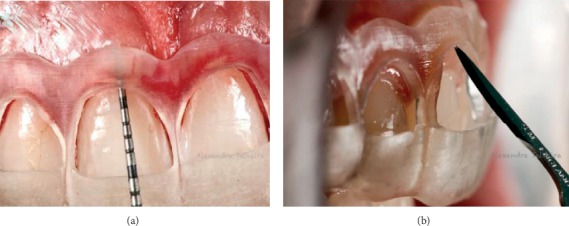
(a) CEJ was located. (b) Primary internal bevel incision.

**Figure 7:**
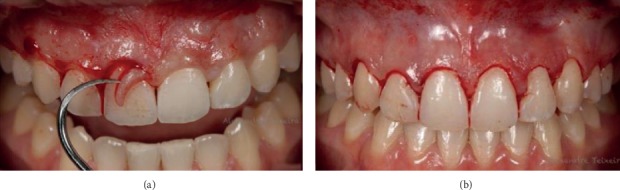
(a) Gingival collar removal. (b) Gingival collar removed.

In the surgical stage, firstly, extraoral asepsis was performed with a 10% povidone-iodine (PVPI) solution and the patient was asked to rinse the mouth with 0.12% chlorhexidine digluconate for one minute. Subsequently, infiltrative local anesthesia with 4% articaine (Nova DFL, Industry and Commerce S.A, Rio de Janeiro, Brazil) was administered at the bottom of the maxillary vestibule. Using a millimeter probe (Hu-Friedy, Chicago, USA), the CEJ was located and compared with the delimitation of the surgical guide positioned in the mouth (Figures 6(a)). The primary internal bevel incision was performed with a 15C blade (Figure 6(b)) (Swann-Morton, England), followed by intrasulcular incisions for collar removal (Figures 7(a) and 7(b)).

**Figure 8:**
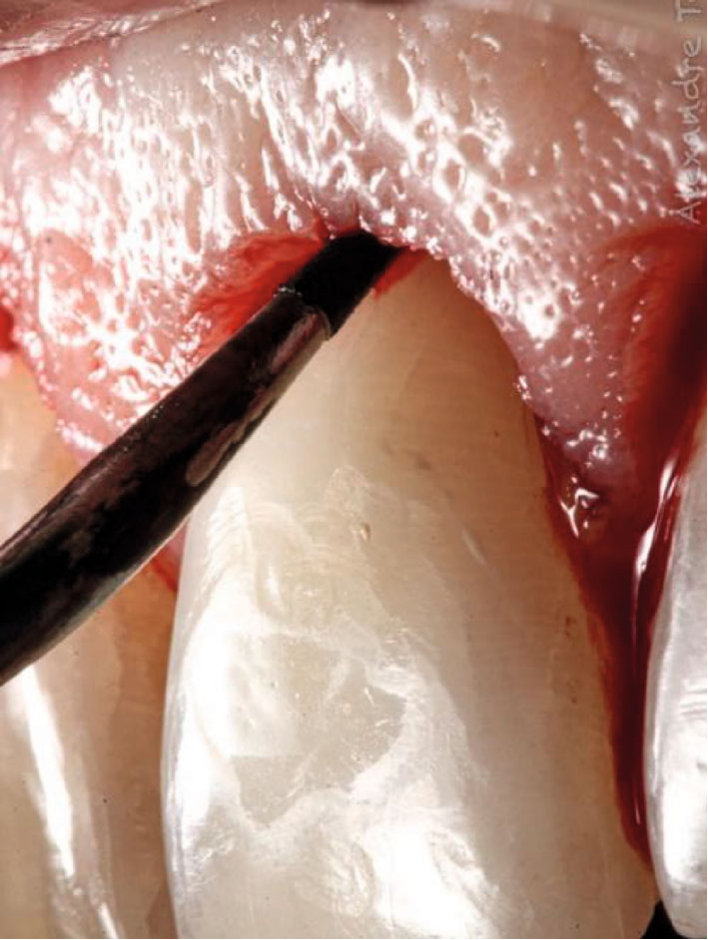
Specific insertion for tunneled osteotomy with the piezoelectric device, where the cut is only at the tip to avoid damage to the tooth root.

**Figure 9:**
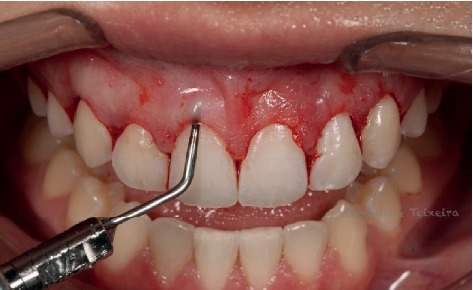
Tunneled osteotomy with a piezoelectric.

Gingival tunneling was performed with the aid of a tunneling instrument, and tunneled osteotomy (tunneling) was performed with a piezoelectric device with a specific insert for this purpose. The frequency of the piezoelectric device is regulated for bone, and not for cementum and dentin, thus preventing damage to the tooth root and crown. Also, a slight inclination of the insert is made so that it touches the bone (Figure 8). Its tips measuring 3 mm were inserted into the gingival sulcus to reestablish biologic width (Figure 9). The patient was medicated for pain and edema control with paracetamol 750 mg and ibuprofen 600 mg every 8 hours for 3 days.

**Figure 10:**
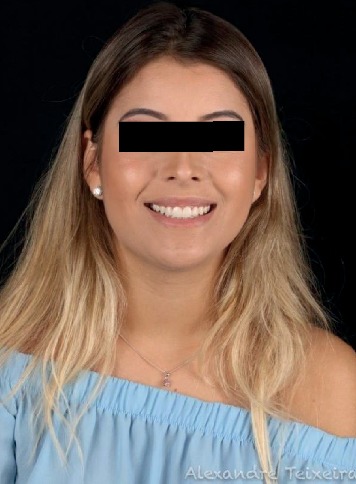
60 days after surgery.

In the immediate postoperative period, the patient presented better gingival contour and was completely satisfied with the treatment at the 60-day evaluation (Figure 10). Whitening was performed after 90 days of surgery, and class III resins were subsequently corrected to harmonize the smile.

## 3. Discussion

This case report reinforces the interrelationship of periodontics and aesthetic dentistry, considering that both require a multidisciplinary treatment plan in gummy smile patients. Preapproved reverse planning with the diagnostic mockup increased patient confidence. The preview of the result helped in aesthetic and functional evaluation. In the search for smile aesthetics, the correction of the gingival contour before rehabilitation is crucial to obtain better results, and a differential diagnosis is needed to proceed with the specific treatment plan for each etiology [3]. In this case, digital planning supported the obtained results.

Altered passive eruption (APE) is a condition in which the gingiva does not migrate to its final position in the apical direction. In the present case report, clinical examination and dental gingival tomography revealed that APE was the etiological factor. However, a gum exposure of less than 3 mm when smiling is considered acceptable. In an ideal smile, 1 mm to 2 mm of the gum is exposed when the upper lip moves apically [3]. For gummy smile correction, the length of the clinical crown is increased to reduce the amount of exposed gum and increase the crown height to follow the upper lip line [13]. Most cases require osteoplasty to reduce marginal bone and restore the biologic width, as described above. When the gingival margin is too close to the CEJ, there is not enough space for the establishment of the biological space [14], leading to a greater likelihood of gingival growth recurrence after gingival contouring surgery [15]. Therefore, studies have demonstrated a gingivectomy technique initiated by an internal bevel incision and followed by an intrasulcular incision to remove the collar. Subsequently, the flap is raised to the mucogingival line for osteotomy and osteoplasty to reestablish the biologic width [12, 15–19]. Digital treatment planning enables the clinician to plan the amount of osteotomy needed on each tooth to maintain the biologic width.

Silva et al. [23] described a “flapless” approach with gingival sulcus osteotomy using microchisels in the treatment of a gummy smile with altered passive eruption. The results are aesthetically favorable and predictable in patients with thin or intermediate gingival biotypes and a keratinized gingival width of at least 3 mm. Flapless surgery is contraindicated in patients with a thick gingival phenotype because a precise and well-designed osteotomy is needed to obtain a better adaptation of the soft tissues in the cervical region and harmonize the papillae [20]. In our case, the thick periodontal biotype was conducive to the flapless technique. However, piezoelectric surgery was used instead of chisels for osteotomy. The benefits of this method include better soft tissue healing, reduced surgical time, no suture, and less postoperative discomfort. Due to the characteristics of the piezosurgery, also known as piezoelectric bone surgery, the osteotomy technique has become more predictable. The microvibrations allow a precise cut of only mineralized structures without damaging soft tissues, even in case of accidental contact. It also provides a bloodless site and decreases undesirable inflammatory responses such as edema and pain [21, 22]. Moreover, it is important to note that we used a piezoelectric with a specific periodontal tip with a 3 mm marking that guided the clinician to a more accurate and safer osteotomy.

Periodontics walks in a digital stream. Technology resources such as dental gingival computed tomography, facial scanning, surgical guides, stereolithography, and the piezoelectric technique have enriched treatment planning and surgery, from the initial clinical examination to the outcome. Clinical probing has been the standard method to localize the CEJ [17]. However, three-dimensional computed tomography with the use of labial and lingual retractors for image acquisition merged with the STF file (generated through intraoral scanning) in specific software was a less invasive method. This provided visualization of the periodontal structures and their relationship to the hard and soft tissues. Besides, it provides measurements of the relationship of the gingival margin to the bone crest, the bone crest to the CEJ, and the CEJ to the gingival margin, buccal and lingual gingival thickness, bone density, and total biologic width [14].

Reverse planning is a concept in dentistry that allows the clinician to visualize the outcome before initiating treatment. In this case, the patient was extremely pleased when the digital wax-up was presented to her. Through the innovations of digital workflow, it has become possible to refine this technique and use it in periodontics with specific software. Before starting the treatment, patients can see themselves using digital wax-ups (3D Planning, BDS) based on measurements and proportions obtained after aesthetic-facial and intrabuccal analysis. Subsequently, the clinician can use the “PrintedMockup” (digital waxing, printed on resin) to describe the severity of the case and the desired corrections to the patients. They can discuss the prognosis together and reach a consensus to proceed with the ideal treatment plan. We noticed that the patient was deeply moved when the guide was placed in her mouth, which contributed positively to successful treatment.

## 4. Conclusion

We can conclude that guided periodontal surgery for the treatment of a gummy smile using piezoelectric digital planning favors postoperative results, making treatment more predictable and consistent with patient expectations. The procedure is faster, more accurate, and safer.
